# Zinc Finger Recombinases with Adaptable DNA Sequence Specificity

**DOI:** 10.1371/journal.pone.0019537

**Published:** 2011-04-29

**Authors:** Chris Proudfoot, Arlene L. McPherson, Andreas F. Kolb, W. Marshall Stark

**Affiliations:** 1 College of Medical, Veterinary and Life Sciences, University of Glasgow, Glasgow, Scotland, United Kingdom; 2 Nutrition and Epigenetics Group, Life Long Health Division, Rowett Institute of Nutrition and Health, University of Aberdeen, Aberdeen, Scotland, United Kingdom; University of Minnesota, United States of America

## Abstract

Site-specific recombinases have become essential tools in genetics and molecular biology for the precise excision or integration of DNA sequences. However, their utility is currently limited to circumstances where the sites recognized by the recombinase enzyme have been introduced into the DNA being manipulated, or natural ‘pseudosites’ are already present. Many new applications would become feasible if recombinase activity could be targeted to chosen sequences in natural genomic DNA. Here we demonstrate efficient site-specific recombination at several sequences taken from a 1.9 kilobasepair locus of biotechnological interest (in the bovine β-casein gene), mediated by zinc finger recombinases (ZFRs), chimaeric enzymes with linked zinc finger (DNA recognition) and recombinase (catalytic) domains. In the "Z-sites" tested here, 22 bp casein gene sequences are flanked by 9 bp motifs recognized by zinc finger domains. Asymmetric Z-sites were recombined by the concomitant action of two ZFRs with different zinc finger DNA-binding specificities, and could be recombined with a heterologous site in the presence of a third recombinase. Our results show that engineered ZFRs may be designed to promote site-specific recombination at many natural DNA sequences.

## Introduction

The vast amount of genomic sequence data now available has led to an increasing awareness of the far-reaching potential and implications of ‘genomic surgery’-that is, the locus-specific insertion, deletion or replacement of defined DNA sequences. Genomic surgery methods could transform the treatment of genetic diseases and lead to more sophisticated use of genetic manipulation in biotechnology and experimental genetics. However, development of the molecular ‘surgical instruments’ to break and rejoin DNA strands at sequences of our choice and thus bring about desired genetic rearrangements is still at a primitive stage [1], [2]. The greatest successes in this field to date have been achieved with zinc finger nucleases (ZFNs), which comprise an endonuclease domain fused to a zinc finger DNA-binding domain. ZFN heterodimers can introduce a double-strand break (DSB) at targets with appropriately spaced binding sites for two different zinc finger domains [3], [4], potentially leading to high-frequency homologous recombination or mutagenic DNA repair at the target locus. Current advanced methods for creation of altered-specificity zinc finger domains [4] allow ZFNs to be designed to target a wide range of natural sequences. However, finalization of the desired genetic changes requires repair or recombination at the ZFN-induced DSB mediated by endogenous enzymes. Inefficient or aberrant operation of these processes often results in low levels of gene modification or undesired sequence changes [5]. Similar limitations apply to the use of all other agents that simply introduce site-specific DSBs.

**Figure 1:**
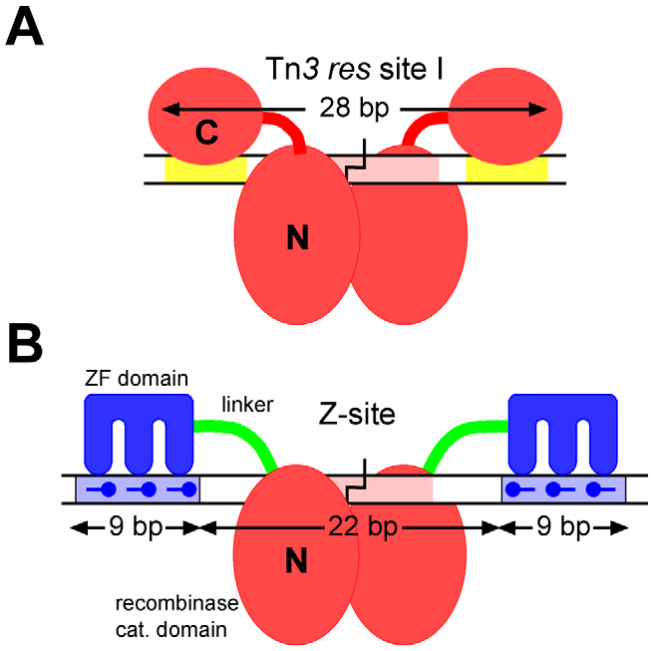
Design of ZFRs, Z-sites and test substrates. (**A**) Cartoon showing a Tn*3* resolvase dimer bound to *res* site I. The red ovals represent the N-terminal (catalytic) and C-terminal (DNA-binding) domains. The motifs recognized by the C-terminal domains are yellow, and the central 12 bp segment (pink) is contacted by the catalytic domains.(**B**) Cartoon showing a ZFR dimer bound to a Z-site. The zinc finger domains are blue, the motifs they recognize are pale blue, and the ZFR linker peptide is green. Other features are as in **A**.

In contrast, site-specific recombinases possess all the enzyme functions required to bring about efficient, precise integration, deletion or inversion of defined DNA segments [6], and might be very effective instruments for genomic surgery if their target specificity could be altered at will [1]. Already, directed evolution methods have been applied to create recombinase variants with activity at certain genomic sites which bear some resemblance to the target site of the original recombinase [7], [8]. However, a more incisive strategy is to create zinc finger recombinases (ZFRs) analogous to ZFNs, which could be re-targeted by changing the DNA recognition specificity of the zinc finger domains. Serine recombinases of the resolvase-invertase group, such as the well characterized Tn*3* and γδ resolvases and the Hin and Gin invertases, are especially attractive as starting points for this approach because of their modular structure, with autonomous catalytic and DNA-binding domains (Figure 1A) [6]. The catalytic domains of these recombinases might therefore be predicted to function when linked to different (zinc finger) DNA-binding domains. However, recombination activity by natural serine resolvases and invertases is strictly dependent on the presence of complex regulatory DNA sequences containing binding sites for extra recombinase subunits and/or accessory proteins [6], and the natural catalytic domains are inactive in the absence of these features. The development of ZFRs was only feasible following the isolation of ‘activated’ recombinase mutants which do not require any accessory factors, and recombine short dimer-binding sites [9]–[12]. In previous work from this laboratory, ZFRs (previously called Z-resolvases) were made by linking the catalytic domain of a Tn*3* resolvase activated mutant to a zinc finger (Zif268) DNA-binding domain. These ZFRs promote efficient recombination at Z-sites (Figure 1B), which have a central sequence acted upon by the catalytic domains (optimally 22 bp), immediately flanked by 9 bp motifs recognized by the Zif268 domains [13]. ZFRs, like ZFNs, have the potential to target many new sequences, being limited in principle only by the range of sequences for which specific zinc finger domains can be made and by the sequence selectivity of the catalytic domain [1]. Many natural serine recombinases with distinct sequence specificities are known [14], and their catalytic domains could be co-opted to make ZFRs if suitable activated mutants were available [1]. ZFRs with catalytic domains from Tn*3* resolvase and Gin invertase have been demonstrated to be active in human cell lines [15], [16], illustrating the potential applicability of these enzymes in mammals and other eukaryotic organisms.

The Tn*3*/γδ resolvase catalytic domains contact the recombination crossover site (site I) in the minor groove over about 12 bp (Figure 1A) [17], [18]. Some mutations of this 12 bp sequence strongly inhibit recombination, whereas others do not [19]. The structural basis for this partial specificity and the consensus sequence for the resolvase catalytic domain are still unclear. In our earlier experiments with ZFRs the Z-site central sequence was similar to that of site I, to optimize catalytic domain interactions [13]. However, the specificity of the catalytic domain would need to be altered or reduced in order to target most natural sequences which do not resemble site I. Here, we prove the wide applicability of ZFRs by showing that a number of sequences within a short (<2 kbp) genomic locus of biotechnological interest are suitable as recombination targets for ZFRs with evolved broad specificity catalytic domains. Furthermore, pairs of evolved ZFRs with different DNA-binding specificities can cooperate to recombine asymmetric Z-sites, and, in a model for targeted gene integration, an asymmetric Z-site can recombine with a partner site that binds a third recombinase. Our results highlight the great potential of ZFRs as next-generation tools for genomic surgery.

## Materials and Methods

### ZFR expression plasmids and recombination substrate plasmids

Expression plasmids for ZFRs or the NM variant of Tn*3* resolvase [10] were of two types. Plasmids with a pBR322 origin of replication (pβZFR) were similar in construction to those previously described [13]. A resolvase expression plasmid with a p15a origin of replication (pEK76) was derived from pACYC184 [20], by insertion of an SspI-EcoRV resolvase-encoding fragment from pAT5 [21] into the pACYC184 AvaI site. ZFR expression plasmids (pαZFR) were then created by insertion of ORF-containing fragments between unique NdeI and Asp718 sites in pEK76. In order to allow simultaneous expression of three recombinases in an *E. coli* strain, two further expression plasmids pαZFR^AB^320 and pβZFR^AB^320 were made, which have the reading frames and translational start signals for ZFR^A^320 and ZFR^B^320 (described in the Results section) in tandem.

The ‘progenitor’ ZFR used here (ZFR300) consists of the Tn*3* resolvase catalytic domain (residues 1–148) containing the seven mutations R2A E56K G101S D102Y M103I Q105L V107F, followed by the 2-amino acid linker TS, then the Zif268 DNA-binding domain starting at residue E2 (ERPY...). The codons for the TS linker introduce a unique SpeI restriction site. Some of the reasoning behind the design of the ZFR300 amino acid sequence has been reported [13]; further details will be described elsewhere (M. Prorocic *et al.*, unpublished). Mutations in the Zif268 domain of ZFR300 were introduced by cloning oligonucleotides between appropriate restriction sites in a pβZFR plasmid.

**Figure 2:**
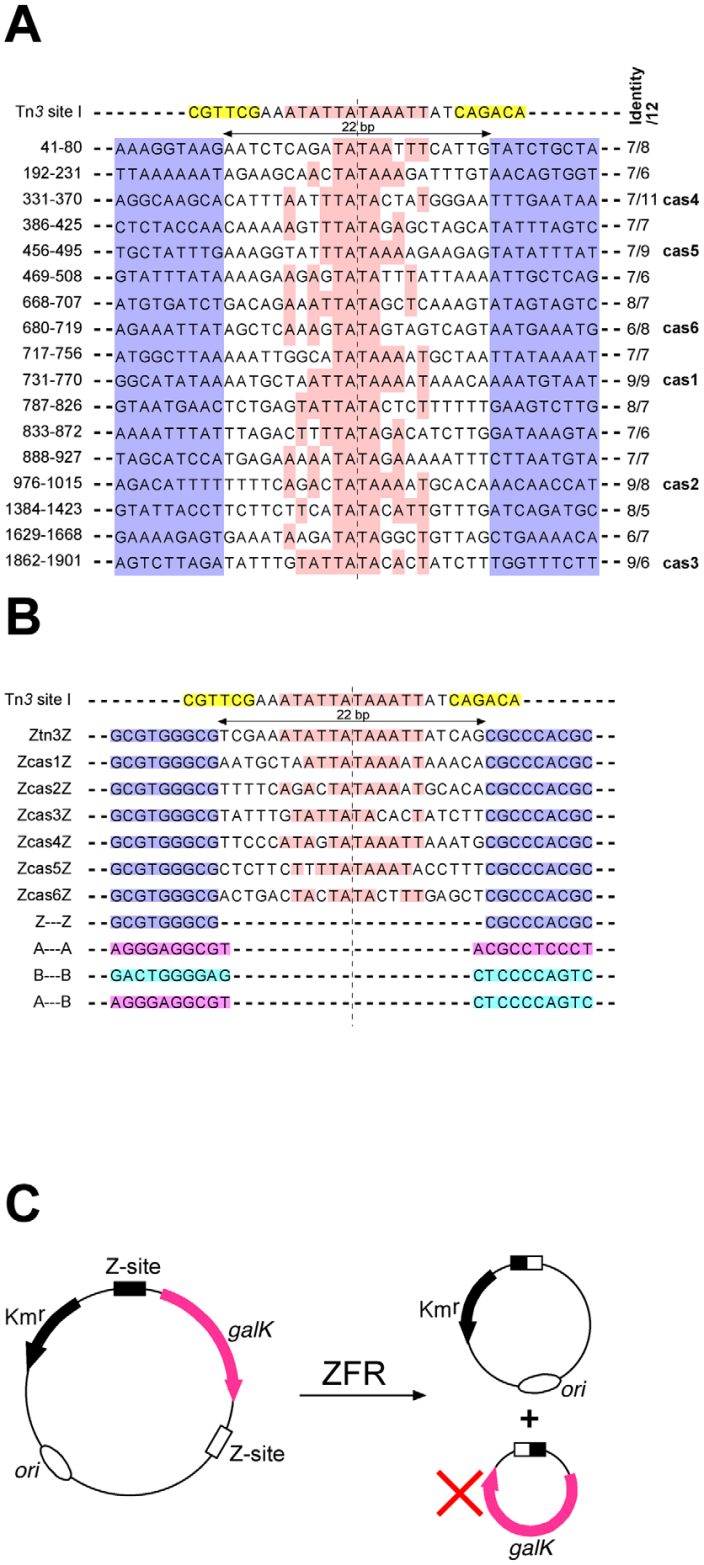
Casein gene sequences, Z-sites, and recombination assay. (**A**) Tn*3 res* site I (top line) and the seventeen TATA-containing sequences from the β-casein gene intron 1 (Genbank accession number X14711), aligned with the central TATA of site I. Sequences are numbered from the start of exon 1. The motifs in site I bound by the resolvase C-terminal domains are highlighted in yellow. Bases identical to site I within the central 12 bp of the casein sequences are highlighted in pink. The column on the right gives the number of bases identical to the central 12 bp of site I (the number before the slash is for the alignment shown, and the number after the slash is for the sequence aligned in the opposite orientation). The six ‘cas’ sequences, whose central 22 bp sequences were used in the recombination sites analysed in this study, are indicated. The 9 bp sequences flanking the central 22 bp are also shown (highlighted in blue); these sequences would be bound by the ZFR zinc finger domains if the genomic casein gene sequences were to be targeted as Z-sites (see Figure 1B). (**B**) Sequences of site I and Z-sites. The Z-site motifs recognized by the Zif268 domains are highlighted pale blue. The central 22 bp sequences of the ZcasZ sites are from the casein gene sequences shown in part **A**. Identities of the sequences to the central 12 bp of site I are highlighted pink. Note that the ZcasZ sequences are aligned here to maximize their matches with site I, so some are in the opposite orientation from part A. Motifs recognized by mutant Zif268 domains ZifA and ZifB are highlighted in magenta and cyan respectively. See text for further details. (**C**) Z-site substrate plasmids and colony colour assay. Recombination between the two Z-sites (boxes) deletes the *galK* gene, causing colonies to be pale rather than red on MacConkey-galactose indicator plates.

The sequences of the Z-sites used here are shown in Figure 2B. Recombination substrate plasmids, with two Z-sites flanking a *galK* marker gene, were analogous to those described [13]. Full details of the plasmid constructions and sequences are available on request.

### Mutagenesis, and construction of mutant libraries

The entire ZFR reading frame in pβZFR300 (or variants thereof) was mutagenized by PCR with the addition of modified dNTPs, as described [10]. The primers were 22F (5′-CGCCAGGGTTTTCCCAGTCACG-3′) and 23R (5′-TCACACAGGAAACAGCTATGACC-3′), and either dPTP or d(8-oxoG)TP [22] was added at 10% of the total dNTP concentration. A sample of the PCR product was re-amplified without mutagenic dNTPs, so that the DNA fragments to be cloned did not contain unnatural bases. Following purification, the cleaned-up PCR product was digested with NdeI and SpeI, and the 447 bp mutagenized fragment (encoding the resolvase catalytic domain) was ligated to the NdeI-SpeI vector fragment of pβZFR300. Libraries of mutant expression plasmids (library size ∼10^5^) were created by transformation of competent *E. coli* DS941 cells with the ligation products and preparation of plasmid DNA from colonies on agar plates, as described [10].

### Recombination assays, screening libraries and isolation of mutants

*E. coli* colony colour assays of recombination were as described [13]; briefly, transformants were selected on MacConkey-galactose indicator plates (MacConkey agar base (Difco) supplemented with 1% galactose, kanamycin (to select for the substrate plasmid or its resolution product), and ampicillin (to select for pβZFR expression plasmids) and/or chloramphenicol (to select for pαZFR expression plasmids). Pale coloured (*galK* ¯) colonies indicate recombination (resolution) proficiency, whereas red (*galK* ^+^) colonies indicate lack of resolution.

To screen for recombination-proficient ZFR mutants, DS941 cells containing a recombination substrate plasmid were transformed with a library of mutant expression plasmids. Aliquots of the transformants were selected on MacConkey plates (aiming for ∼1000 colonies per plate; 30–60 plates). Pale-coloured colonies were picked and were streaked on MacConkey plates to confirm the colony colour. Plasmid DNA purified from positive isolates was used to transform the same DS941-substrate plasmid strain. If the transformant colonies on MacConkey plates were all pale-coloured, the ZFR was deemed to be a recombination-proficient mutant, so the expression plasmid was isolated and the ZFR reading frame was sequenced.

**Figure 3:**
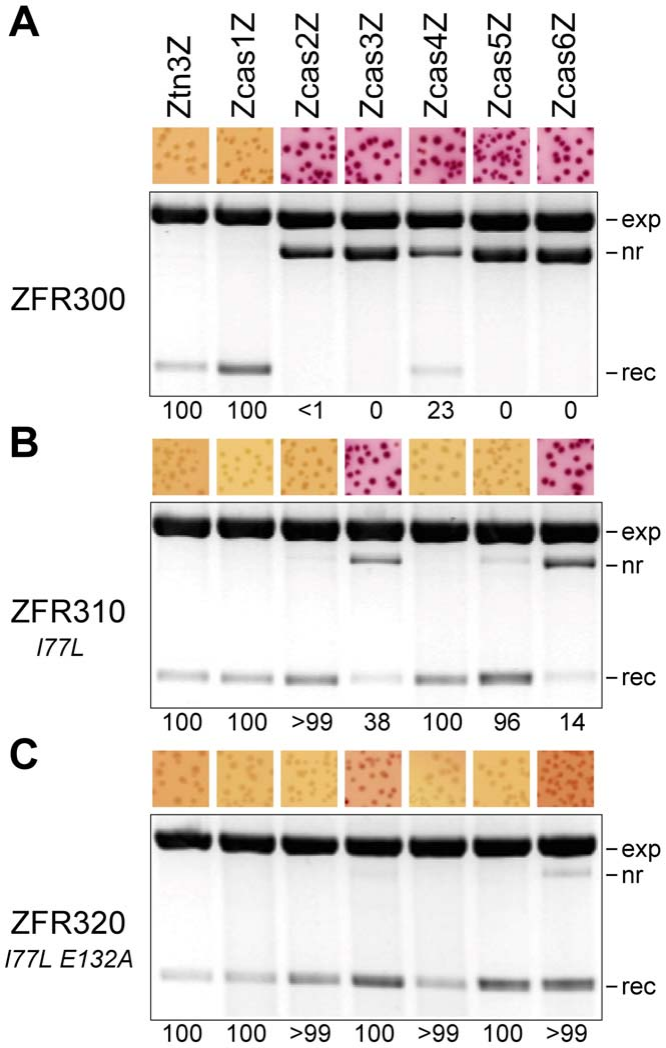
Selection of ZFRs to recombine casein gene sequences. (**A**) Activity of the progenitor ZFR (ZFR300) on Z-sites with central sequences from Tn*3 res* site I or the casein gene intron 1. The substrate plasmids each contained two identical Z-sites as indicated above the gel lanes. The numbers below each lane represent the percentage of recombinant plasmids in the recovered DNA (see Materials and Methods). Images of the ethidium-stained gels are greyscale-inverted, and a uniform background subtraction has been applied. A representative sector of a plate from the corresponding MacConkey-galactose assay is shown above each lane. Annotation: exp, ZFR expression plasmid; nr, non-recombinant substrate plasmid; rec, recombinant (*galK* ^−^) plasmid. (**B**) Activity of the first round mutant I77L (ZFR310) on the same substrates as in **A**. (**C**) Activity of the second round double mutant I77L E132A (ZFR320) on the same substrates as in **A**.

**Figure 4:**
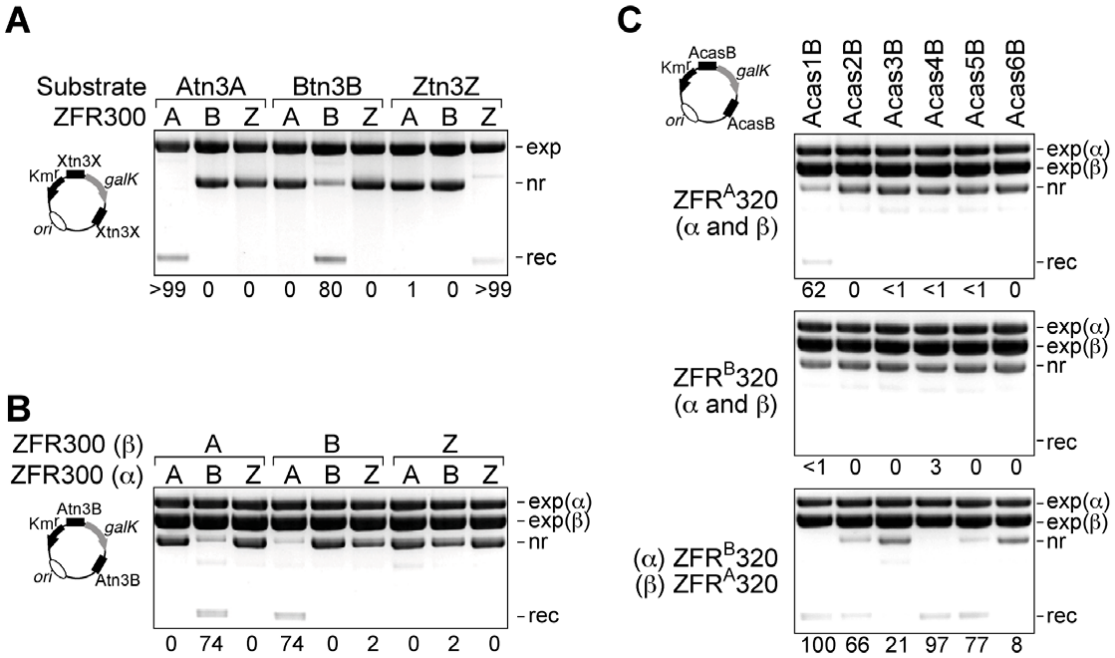
Recombination of asymmetric Z-sites. **(A)** Altered-specificity zinc finger domains target ZFRs to their cognate Z-sites. Substrates containing two identical Z-sites, with either A, B, or Z flanking motifs (Atn3A, Btn3B, Ztn3Z; Figure 2B), were recombined by ZFR300 variants with alternative zinc finger domains as indicated immediately above each lane. (**B**) Efficient recombination at asymmetric (Atn3B) Z-sites requires two ZFR300 variants, with ZifA and ZifB specificity. The letters above each lane indicate the binding domains borne by the expressed ZFRs, with specificity for either Zif268 (Z), ZifA (A), or ZifB (B) motifs. exp(α), pACYC184-based ZFR expression plasmid (pαZFR); exp(β), pBR322-based ZFR expression plasmid (pβZFR). (**C**) Efficient recombination of AcasB site substrates requires co-expression of ZFR^A^320 and ZFR^B^320.

**Figure 5:**
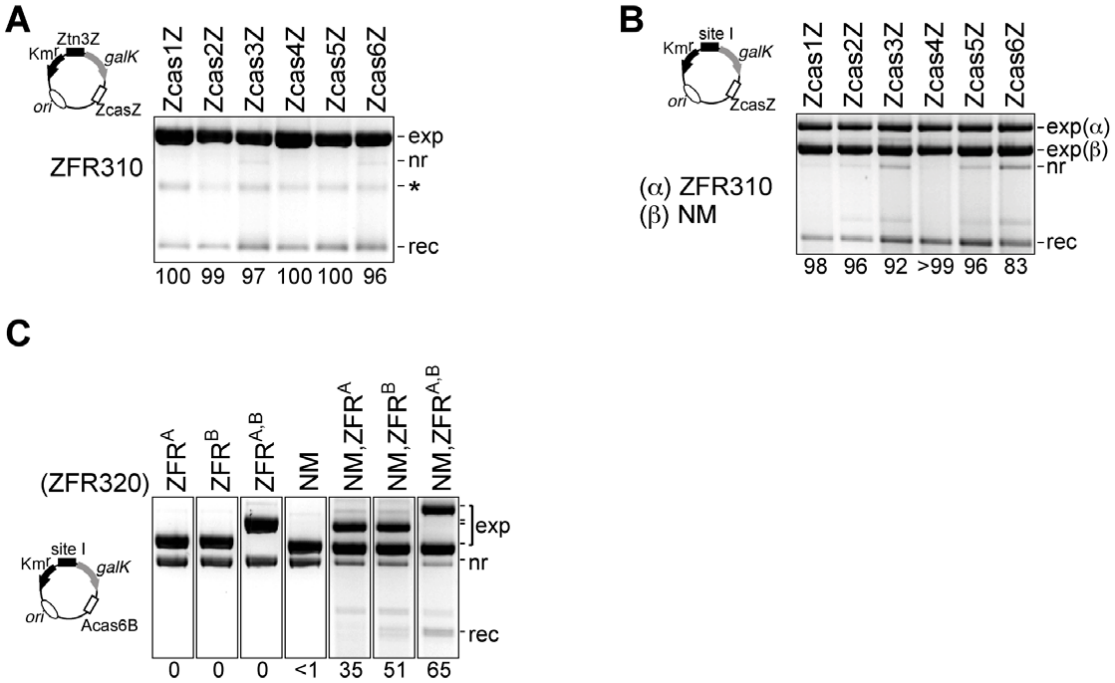
Recombination between non-identical sites. (**A**) Substrates containing a Ztn3Z site and a ZcasZ site are recombined by ZFR310. The annotation is as in the previous Figures. The band marked with an asterisk is thought to be single-stranded expression plasmid DNA. (**B**) Substrates containing a ZcasZ site and Tn*3 res* site I are recombined when ZFR310 and NM resolvase are co-expressed. (**C**) A substrate containing an Acas6B site and Tn*3 res* site I is recombined most efficiently when NM resolvase, ZFR^A^320 and ZFR^B^320 are co-expressed.

To analyse the DNA products of recombination, strains containing a substrate plasmid were transformed with a ZFR expression plasmid or, for experiments with two or three recombinases, co-transformed with two expression plasmids having compatible origins of replication (pαZFR and pβZFR; see above). Transformants were grown with appropriate antibiotic selection on L-agar plates. Following incubation at 37°C, cells were washed from the plates with L-broth, and plasmid DNA was purified from the cells using a Qiagen miniprep kit. The DNA was visualized by ethidium staining after 1.2% agarose gel electrophoresis. To quantify the extent of recombination (leading to deletion of the *galK* gene) more accurately, the recovered plasmid DNA was used to transform the strain DS941, test plasmid-containing transformants were selected on MacConkey agar plates with kanamycin, and the percentage of pale (*galK* ¯) colonies (from a total of >100) was determined. In Figures 3, 4, and 5, a value of 0 indicates that all colonies were red (*galK* ^+^), 100 indicates that all were pale, and <1 (or >99) indicates that there were a few pale (or red) colonies.

## Results

### Experimental design

The 1930 bp first intron of the bovine β-casein gene was chosen as a target locus with potential biotechnological utility; a transgene integrated at this locus could be regulated by the β-casein promoter, giving high-level expression of a transgenic protein in milk. Our aim here was to identify sequences within this intron that would be suitable targets for ZFR-mediated integration. Analysis of the intron sequence revealed 17 occurrences of the TATA motif which is found at the centre of Tn*3 res* site I, and which might be optimal for resolvase activity [19] (Figure 2A). Six of these potential target sequences that were most similar to site I over the 12 bp centred on the TATA motif (with at least 8 out of 12 basepairs identical in one orientation; Figure 2A) were selected for further analysis. ‘ZcasZ’ sites were made, each comprising 22 bp of a casein gene intron sequence flanked by 9 bp motifs recognized by the Zif268 zinc finger domain (Figure 2B). A control Ztn3Z site with Tn*3 res* site I central sequence was also made. Plasmids were then constructed in which two identical Z-sites are separated by a *galK* marker gene (Figure 2C). If ZFR-mediated recombination (resolution) in *E. coli* cells deletes *galK*, the colonies on MacConkey-galactose indicator plates are pale, whereas *galK* ^+^ colonies are red [10]. ZFR-mediated recombination can also result in inversion of the orientation of the *galK* segment, but this outcome is not detected by the assays used here.

### Selection of active ZFR variants

We began by analysing recombination of the substrates by an already available ZFR. ZFR300 consists of the catalytic domain of an activated Tn3 resolvase variant, linked to the Zif268 zinc finger DNA-binding domain (see Materials and Methods). E. coli cells containing a substrate plasmid were transformed with a ZFR300 expression plasmid. Recombination activity was detected by colony colour on MacConkey indicator plates, and then by electrophoretic analysis of plasmid DNA recovered from the cultured cells. The Ztn3Z substrate was resolved completely by ZFR300, as expected. Encouragingly, two of the six ZcasZ substrates were also recombined; Zcas1Z (complete resolution) and Zcas4Z (incomplete resolution) (Figure 3A).

To find ZFR variants that would promote recombination at the four refractory ZcasZ sites, we subjected the entire catalytic domain to random mutagenesis using two error-prone PCR protocols, and made libraries of plasmids expressing mutant ZFRs. Screens of the libraries using Zcas2Z and Zcas5Z substrate plasmids yielded several active mutants (Table 1), whereas no mutants were recovered from screens with the Zcas3Z and Zcas6Z plasmids. Recovery of DNA from the cells showed that one mutant (ZFR310) with the single conservative change I77L had significant recombination activity on all six ZcasZ substrates, although Zcas3Z and Zcas6Z substrates still gave only red colonies (Figure 3B).

**Table 1:** Mutant ZFRs selected for recombination of Zcas2Z or Zcas5Z sites.

		Z Site						
z1st Round Mutations	No.	Ztn3Z	Zcas1Z	Zcas2Z	Zcas3Z	Zcas4Z	Zcas5Z	Zcas6Z
**ZFR300**		W	W	R	R	R	R	R
**I77L** (ZFR310)	8	W	W	**W**	R	W	**W**	R
F83L F107L	3	W	W	R	R	W	**R**	R
I3T D95E	1	W	W	M	R	W	**M**	R
G70S F107L	1	W	W	**M**	R	W	W	R
E57G A89V	1	W	W	W	R	W	**R**	R
K37R D44N	1	W	W	**W**	R	W	W	R
I97V I103T F107L	1	W	W	**W**	R	W	W	M
G70S	/	n.d.	W	R	R	M	R	R
F107L	/	n.d.	W	R	R	W	R	R
E57G	/	n.d.	W	R	R	R	R	R
A89V	/	n.d.	W	R	R	W	R	R

The Table shows mutants isolated from libraries of mutagenized ZFR300. The left-hand column gives the mutations, and the next column indicates the number of independent isolates. The other columns show the phenotype of each mutant in the MacConkey agar colony colour assay, using substrates with two identical ZcasZ sites as indicated. W, ‘white’ (pale-coloured) colonies; R, red colonies; M, mixtures of pale and red colonies. The letters in bold show the ZcasZ substrate (Zcas2Z and/or Zcas5Z) that was used in the screen from which the mutant was isolated. The four point mutants shown below the thick line were isolated by cloning appropriate fragments from the originally isolated multiple mutant (above the line). n.d., not done.

The mutagenesis-screening procedure was then repeated, using ZFR310 as the template for mutagenesis, and the Zcas6Z test substrate (which gave the lowest level of recombination with ZFR310). Several active mutants were isolated which were shown to promote efficient recombination of the Ztn3Z and all six ZcasZ test substrates (Table 2; illustrated for the double mutant I77L E132A (ZFR320) in Figure 3C). The mutations thus broaden rather than switch specificity. However, selectivity is not completely abolished, and the mutants do not recombine some Z-sites with central sequences that are very unlike site I. For example, ZFR320 did not recombine a Z-site with the central sequence of the recombination site for Sin resolvase [23] (data not shown).

**Table 2:** Mutant ZFRs selected for recombination of Zcas6Z sites.

		Z Site						
2nd Round Mutations	No.	Ztn3Z	Zcas1Z	Zcas2Z	Zcas3Z	Zcas4Z	Zcas5Z	Zcas6Z
**ZFR310**		W	W	W	R	W	W	R
N31D M53R N127K	1	W	W	W	W	W	W	**W**
I3P V108A	1	W	W	W	W	W	W	**W**
L135R	2	W	W	W	W	W	W	**W**
S12R D17E	1	W	W	W	W	W	W	**W**
S12R I103V	1	W	W	W	W	W	W	**W**
**E132A** (ZFR320)	5	W	W	W	W	W	W	**W**
I3L L135R	1	W	W	W	W	W	W	**W**
I3S E132A	1	W	W	W	W	W	W	**W**
N127H E132A	1	W	W	W	W	W	W	**W**
I3S D25A F83L	1	W	W	W	W	W	W	**W**

The Table shows the ‘second round’ mutants isolated from screens of ZFR310 mutant libraries (ZFR310 is ZFR300 with the mutation I77L), using a test substrate containing two Zcas6Z sites. The left-hand column gives the mutations, and the next column indicates the number of independent isolates. The other columns show the phenotype of each mutant in the MacConkey agar colony colour assay, using substrates with two identical ZcasZ sites as indicated. W, ‘white’ (pale-coloured) colonies; R, red colonies; M, mixtures of pale and red colonies.

### Recombination of asymmetric Z-sites mediated by pairs of ZFRs

Next, we demonstrated that ZFR activity can be targeted to different Z-sites by substitution of the Zif268 zinc finger domain with altered-specificity variants. The ZFR300-coding sequence was modified so as to replace the Zif268 domain with ZifA or ZifB, synthetic variants with published sequences and target sites (Figure 2B) [24]. The resulting recombinases ZFR^A^300 and ZFR^B^300 were tested along with ZFR300, on substrate plasmids with pairs of Ztn3Z, Atn3A, or Btn3B Z-sites (the sites are shown in Figure 2B). Each substrate was recombined by the ZFR whose zinc finger domain was specific for the Z-site sequences, but not by the ZFRs with different specificity (Figure 4A).

Targeting of a natural asymmetric sequence (such as the chosen casein gene sequences) would require two ZFRs which would interact on the site to form a heterodimer, one ZFR binding to each end of the site. Previous work from our laboratory showed that activated resolvase mutants assemble DNA-bound dimers and synaptic tetramers from solution monomers [11], so we predicted that ZFRs would behave similarly and readily form DNA-bound heterodimers. We made test substrates with pairs of Atn3B or AcasB Z-sites (each of these sites has one motif recognized by ZifA and one by ZifB; Figure 2B). To test for recombination, an E. coli strain containing a substrate was transformed with two compatible plasmids, each expressing one ZFR variant. Efficient recombination of the Atn3B substrate was observed only in the presence of both ZFR^A^300 and ZFR^B^300 (Figure 4B). The AcasB substrates were tested with ZFR^A^320 and ZFR^B^320, which have the broadest-specificity evolved catalytic domain. All the substrates were recombined when both ZFR^A^320 and ZFR^B^320 were expressed; the extent of recombination (i.e. resolution of the test plasmid) ranged from 100% (Acas1B) down to 8% (Acas6B) (Figure 4C).

### ZFR-mediated recombination between non-identical sites

For transgene integration at any chosen genomic locus, only one of the recombining sites (the genomic one) must be targeted by ‘designer’ ZFRs. The other site (on the transgenic DNA) could be acted on by any recombinase with a compatible catalytic domain, and could be optimized for integration efficiency and specificity. To model this scenario, we made substrates with non-identical pairs of sites. The Ztn3Z site contains at its centre 22 bp of the natural target sequence for our ZFR catalytic domains (Tn*3 res* site I), and a substrate with two of these sites is therefore recombined efficiently (Figure 3A). To analyse recombination between Ztn3Z and each of the ZcasZ sites, we made a set of six ZcasZ×Ztn3Z substrate plasmids. These plasmids were recombined more efficiently than the corresponding ZcasZ×ZcasZ plasmids; in fact, the ‘first round’ mutant ZFR310 (with the single mutation I77L) resolved all of them (Figure 5A), whereas it had failed to resolve the plasmids with pairs of Zcas3Z or Zcas6Z sites (Figure 3B). Then, in order to show that the partner site need not be a Z-site, we made substrates which contained a ZcasZ site paired with Tn*3 res* site I. Recombination of all six ZcasZ×site I substrates was efficient when both an activated Tn*3* resolvase variant (NM resolvase; [11]) and a ZFR (ZFR310) were co-expressed (Figure 5B), whereas NM resolvase alone or ZFR310 alone did not promote recombination (data not shown).

Finally, we tested a substrate designed to model transgene integration as outlined above, where one "genomic" recombination site is targeted by the combined action of two designer ZFRs, and the other "transgene" site is recognized by a third recombinase. For the "genomic" site we chose to use Acas6B, in which the least reactive casein sequence is flanked by motifs recognized by engineered variant ZifA and ZifB domains. The sequence of the Acas6B site is quite unlike that of the natural targets of either Tn*3* resolvase or Zif268. For the "transgene" site we used Tn*3 res* site I, as in the experiments described above. The substrate is cartooned in Figure 5C. Acas6B×site I recombination required the expression of NM resolvase and ZFRs, and was most efficient when ZFR^A^320, ZFR^B^320, and NM resolvase were all present (Figure 5C), although significant recombination also occurred when either one of the ZFR proteins and NM resolvase were co-expressed.

## Discussion

Ideally, if ZFRs are to become widely applicable, recombination activity should be insensitive to the central sequences of the target Z-sites, which interact with the recombinase catalytic domains. A useful comparison may be made with the zinc finger nucleases (ZFNs), whose catalytic domain (from the FokI restriction endonuclease) has low sequence selectivity. However, it is clear that natural serine recombinase catalytic domains contribute significantly to DNA sequence specificity; for example (as noted above), recombination by γδ resolvase is strongly inhibited by some mutations of the target site for its catalytic domains [19]. When we began this project, it was not clear whether the specificity of the resolvase catalytic domain could be reduced to the point where a substantial number of genomic sequences could be targeted effectively. Recently, mutant ZFRs with Tn*3* resolvase- and Gin-derived catalytic domains were shown to promote recombination at some specific non-canonical target sequences [25].

We chose the bovine β-casein gene intron as a potential recombinase target because of the potential biotechnology utility of integration at this locus, not because it was known to contain any sequence resembling a natural recombination site. Six sequences from the 1.9 kbp intron were inserted into Z-sites and shown to be recombined efficiently by ZFRs with evolved Tn*3* resolvase-derived catalytic domains. However, many more sequences in the intron might also be suitable for ZFR-mediated recombination. All the chosen sequences had a central TATA motif like Tn*3 res* site I, and there are 11 more of these in the intron (Figure 2A), but a central TATA is not essential for resolvase activity [19]; 98 other sequences are identical to site I at 8 or more positions of the central 12 bp, and many of these might also support recombination. The range of potential target Z-sites might be extended even further by making ZFRs based on catalytic domains from other serine recombinases with different specificities [1], [14]. Many other important genomic loci are likely to be similarly rich in potential Z-sites.

To promote recombination at a typical asymmetric genomic Z-site, two ZFRs might be expected to be required, each with a ‘designer’ zinc finger domain to recognize one end of the site. An optimal site would therefore have left and right ends suitable for binding by engineered zinc finger domains. However, our experiments show that in some cases, recombination activity at an asymmetric Z-site can be elicited by the expression of only one ZFR (Figure 4C, 5C). We hypothesize that a single ZFR subunit bound to its cognate DNA sequence can recruit a second "non-specific" ZFR subunit to the Z-site by dimerization of the catalytic domains. In general, this phenomenon is undesirable, as it could reduce the achievable sequence specificity of ZFRs. A related phenomenon is evident in the recombination reactions involving two or three recombinases (Figures 4 and 5), which were generally not as efficient as those involving only one type of ZFR. We suspect that this reduced efficiency is because inappropriate recombinase dimers can bind to recombination sites and block recombination. For example, the two recombinases used in the experiment shown in Figure 4C (ZFR^A^320 and ZFR^B^320) can assemble into three different types of dimer (homodimers of ZFR^A^320 and ZFR^B^320, and ZFR^A^320-ZFR^B^320 heterodimer). Only the heterodimer matches the AcasB recombination site, but the homodimers can nevertheless bind to the site as they can recognize the DNA sequence at one end. The scenario with three recombinases, as in Figure 5C, is even more complex. ZFNs, which also recognize asymmetric sites as heterodimers, show similar losses of specificity and efficiency due to homodimerization. The problem has been addressed by introducing mutations in the ZFN catalytic domain that favour on-site formation of the desired heterodimers [26], and a similar approach should be applicable to ZFRs. In other respects, ZFRs could be at least as specific for their targets as ZFNs; in fact, ZFRs might have especially high specificity due to their requirement for synapsis of two sites prior to recombination.

Another feature of our experimental results which should be noted is that the total amount of substrate plasmid and its recombinant product is reduced in the presence of some ZFR variants; see, for example, Figures 3C and 4C. It is possible that some of the plasmid is being lost due to a ZFR activity at the Z-sites (for example, incomplete recombination leading to formation of double-strand breaks, a known property of activated mutant recombinases [11]). Further experiments are necessary to ascertain the causes of this "loss of plasmid" effect and to find ways of minimizing any undesirable outcomes of ZFR activity.

Surprisingly, the first round of selection of ZFR mutants for activity on ZcasZ sites did not yield any mutations of residues that contact or are close to the DNA [17], [18] (Table 1), but some mutations (including I77L) were of residues that we had previously identified as being important for regulation of resolvase's catalytic activity [10]. These mutations might therefore broaden sequence specificity by relaxing the stringency of a regulatory mechanism. However, the second-round mutations (additional to I77L) were of a different set of residues, some of which have sidechains that come close to the DNA where the C-terminal end of the catalytic domain is embedded in the minor groove [17]. The two most commonly isolated mutations, E132A and L135R (Table 2), both increase the positive charge of this part of the protein, perhaps thus increasing its general affinity for negatively charged DNA.

Two general types of designer ZFRs may be envisaged; one type, with evolved low-specificity catalytic domains, is reported here, and is likely to be the most accessible for applications in the immediate future. Alternatively, catalytic domains tailored for high-specificity recognition of a Z-site central sequence might be desirable for applications where it is essential to minimize off-target reactions, such as gene therapy. Successful implementation of this strategy will be more challenging, as it will demand a thorough understanding (currently lacking) of the structural basis for DNA sequence recognition by the catalytic domain. A very recent paper [27] describes progress on this front; the specificities of ZFRs derived from Tn*3* resolvase and Gin invertase were partially switched towards activity on Z-sites with the other's central sequence, by mutagenesis of catalytic domain residues in the C-terminal region of the protein that contacts the DNA minor groove. Only one of the six residues chosen for mutagenesis by this group was also identified in our analysis; N127 (Table 2). The set of residues which can be mutated to broaden specificity might therefore not coincide with the set of residues which detect differences in DNA sequence.

ZFRs could be used to promote transgene integration at a specific locus, or deletion of specific genomic DNA segments. In the general case, deletion between two different, asymmetric sites might require up to four ZFRs, each with DNA-binding specificity for one half-site. However, one very attractive potential application for designer recombinases is to delete the integrated proviral DNA of retroviruses such as HIV by recombination between identical sequences in the Long Terminal Repeats (LTRs) that flank the provirus [8], and this would require only two ZFRs.
